# Probable Mechanisms of Needling Therapies for Myofascial Pain Control

**DOI:** 10.1155/2012/705327

**Published:** 2012-12-31

**Authors:** Li-Wei Chou, Mu-Jung Kao, Jaung-Geng Lin

**Affiliations:** ^1^Department of Physical Medicine and Rehabilitation, China Medical University Hospital, Taichung 40447, Taiwan; ^2^School of Chinese Medicine, College of Chinese Medicine, China Medical University, No. 91 Hsueh-Shih Road, Taichung 40402, Taiwan; ^3^Department of Physical Medicine and Rehabilitation, Yangming Branch, Taipei City Hospital, Taipei 11146, Taiwan; ^4^Department of Physical Therapy and Assistive Technology, National Yang-Ming University, Taipei 11221, Taiwan

## Abstract

Myofascial pain syndrome (MPS) has been defined as a regional pain syndrome characterized by muscle pain caused by myofascial trigger points (MTrPs) clinically. MTrP is defined as the hyperirritable spot in a palpable taut band of skeletal muscle fibers. Appropriate treatment to MTrPs can effectively relieve the clinical pain of MPS. Needling therapies, such as MTrP injection, dry needling, or acupuncture (AcP) can effectively eliminate pain immediately. AcP is probably the first reported technique in treating MPS patients with dry needling based on the Traditional Chinese Medicine (TCM) theory. The possible mechanism of AcP analgesia were studied and published in recent decades. The analgesic effect of AcP is hypothesized to be related to immune, hormonal, and nervous systems. Compared to slow-acting hormonal system, nervous system acts in a faster manner. Given these complexities, AcP analgesia cannot be explained by any single mechanism. There are several principles for selection of acupoints based on the TCM principles: “Ah-Shi” point, proximal or remote acupoints on the meridian, and extra-meridian acupoints. Correlations between acupoints and MTrPs are discussed. Some clinical and animal studies of remote AcP for MTrPs and the possible mechanisms of remote effectiveness are reviewed and discussed.

## 1. Introduction

### 1.1. Myofascial Pain Syndrome (MPS)

Myofascial pain syndrome (MPS) has been defined as a regional pain syndrome characterized by muscle pain caused by myofascial trigger points (MTrPs) [1]. Clinically, MPS includes any pain phenomenon due to activation of latent MTrPs as a consequence of a certain pathological conditions including chronic repetitive minor muscle strain, poor posture, systemic diseases, or neuromusculoskeletal lesions (such as sprain, strain, bursitis, enthesopathy, arthritis, and vertebra disc lesion) [2–4].

### 1.2. Myofascial Trigger Point (MTrP)

MTP is defined by Travell and Simons [1, 5] as the most tender (hyperirritable) spot in a palpable taut band of skeletal muscle fibers. Pressure stimulation of a typical MTrP can elicit pain, referred pain, and local twitch response (LTR) (brisk contraction of muscle fibers in its taut band). The pain elicited by compression of this spot is familiar to the patient as the usual pain complaint (pain recognition) [5]. It has been suggested that “spot tenderness”, “taut band”, and “pain recognition” are the three important criteria for the diagnosis of MTrP, and “referred pain” and “local twitch responses” can be “confirmatory signs” for MTrP diagnosis [6]. 

A myofascial pain patient may have both latent and active MTrPs. Latent MTrPs can be identified in most normal adult skeletal muscles and they are tender, but not painful spontaneously [1]. Active MTrPs are painful spontaneously or in response to movement of the involved muscle. In clinical observation, if a latent MTrP is not appropriately treated or the associated underlying pathological lesion is not eliminated, it can be activated to become an active MTrP, or the pain region may expand to other regions and develop other active MTrPs [4, 5]. The original MTrP is called primary MTrP or key MTrP, and the later developed MTrPs are secondary MTrPs or satellite MTrPs [5]. Inactivation of a key MTrP can subsequently eliminate the satellite MTrPs [5, 7]. It has been hypothesized that there are multiple MTrP loci in an MTrP region [3, 7]. An MTrP locus contains a sensory component (sensitive locus or LTR locus) and a motor component (active locus or spontaneous electrical activity locus). 

### 1.3. Treatment of Myofascial Trigger Point

Appropriate treatment to MTrPs can effectively relieve the clinical pain of MPS. The most important strategy in MPS therapy is treating the underlying etiological lesion that causes the activation of MTrPs [2, 3, 5–8]. If the underlying pathology is not appropriately and completely treated, the MTrP can only be inactivated temporarily and never completely. Conservative treatment, such as appropriate systemic nonsteroidal anti-inflammatory drug (NSAID) or local NSAID gel or patch, thermotherapy, manual therapy, and other physical modalities, should be performed prior to more aggressive therapy, such as local steroid injection, spinal facet joint injection, MTrP injection, dry needling, or acupuncture (AcP), especially for acute lesions or mild lesions [2, 3, 5, 8–10]. It is important to eliminate any perpetuating factors causing persistent existence or recurrence of active MTrPs, and to provide adequate education and home programs to patients to avoid recurrent or chronic pain [5]. 

## 2. Hypothetical Pathophysiological Mechanism of the Myofascail Pain Syndrome

### 2.1. Etiology of Myofascial Trigger Point

There is general agreement that acute muscle overload can activate MTrPs. MTrPs due to muscle overactivity can be easily inactivated after avoidance of overuse or inappropriate use. If an acute lesion is not well controlled (usually due to repeated injury, a severe tissue damage, or inadequate treatment), it can become a chronic lesion with progressive scar tissue formation. This scar tissue may be the major cause of degenerative lesions, which could be a source of MTrP activation in later life [11, 12]. 

Most adults have latent MTrPs in most skeletal muscles that can become active in response to any related lesion in another site. A recent study found that every adult had a hyperirritable spot in the brachioradialis muscle, which could be a latent MTrP, or in the vicinity of the latent MTrP, but no latent MTrPs in children under the age of 1 year [13]. It appears that MTrP circuits in the spinal cord develop in later life when the child is growing up. In a later study, Han and her colleagues [14] found that a child had increased sensitivity at the tendon attachment site and the muscle belly after age of 4 years. They concluded that a child might develop an attachment trigger point and a latent MTrP at the brachioradialis muscle after the aged 4 years. Degeneration may develop at middle age or even younger if previous injury was severe or not treated appropriately. A degenerative tissue is vulnerable to reinjury, especially in the elderly or in a weaker tissue. Therefore, appropriate treatment of an injured soft tissue lesion may be important in avoiding chronic myofascial pain. 

### 2.2. Integrated Hypothesis of MTrP

Integrated hypothesis of MTrP, postulated by Simons and Travell [15], have three essential features (excessive acetylcholine release, sarcomere shortening, and release of sensitizing substances) [16]. An increased acetylcholine release in the neuromuscular junction (motor endplate) can cause an increase of the muscle fiber tension (taut band) that containing an MTrP, and subsequently can cause “energy crisis” with increased metabolism and local ischemia and hypoxia which can induce secretion of sensitizing substances to cause pain. The sensitizing substances can further cause abnormal acetylcholine release so that a vicious cycle is completed [16]. It has been suggested that the MTrPs appear to be located in the endplate zone of the affected muscle fibres, where there was increased spontaneous electrical activity and signs of peripheral sensitization of sensory nerves, as well as the appearance of contractures. Excess microendplate potentials result in a sustained alteration of the membrane potential of the muscle fibres around the endplate, and this may result in dysfunction of endplate, and Ca leak from ryanodine channels in the sarcoplasmic reticulum. This would be consistent with sarcomere shortening in a region close to the endplate [15–17]. 

Irritation or sensitization of nociceptors can cause spontaneous pain. Central sensitization may be the main cause of MTrP activation related to a remote lesion and may also cause spontaneous pain without stimulation to the nociceptors. Nociceptors in an MTrP region connect to a group of dorsal horn cells (sensory neurons) in the spinal cord. These “MTrP related sensory neurons” are responsible for central sensitization and for transmission of pain information to the brain. The neural network with connections among these “MTrP related sensory neurons” is defined as an “MTrP circuit” [2, 8]. An MTrP circuit corresponding for a certain MTrP can also send nerve branches to connect with the other MTrP circuit corresponding to other MTrPs. A latent MTrP may become active if stimuli from peripheral sites are strong enough to trigger the MTrP circuit of this latent MTrP. Most adults have latent MTrPs in most skeletal muscles that may become active in response to any related lesion in another site. The “MTrP Circuit” for an MTrP may be responsible for all MTrP phenomena including pain, referred pain, local twitch response, motor dysfunction, and autonomic phenomena via the spinal cord reflex [4, 18]. 

## 3. Needling Therapy for Myofascial Pain ****Syndrome

### 3.1. Definition of Needling Therapy

Needling therapy means any treatment with needles. “Dry needling” is defined as the penetration of a needle through the skin without introduction of any drug, and “injection” is the procedure of needling therapy with introduction of drugs via an “injection needle” (containing a central hollow). Using a solid needle without central hollow or an injection needle with a central hollow can perform “Dry needling”. Kalichman and Vulfsons [19] suggested that dry needling is a cheap, easy to learn with appropriate training, caring lower risk, and minimally invasive treatment modality.

### 3.2. Injection for Myofascial Pain

The traditional MTrP injection technique (multiple needle insertions) is originally described by Travell and Bobb [20]. The needle is moved in-and-out into different directions to encounter the sensitive loci in an MTrP region. In this way, MTrP pain can usually be eliminated nearly completely immediately after most of those multiple sensitive loci have been injected with a drop of local anesthetic agent on each site. Hong [21] has modified this technique to a fast-movement procedure in order to avoid tissue damage from side movement of needle or the grabbing of needle by an elicited LTR. Later, this new technique has been recommended by Simons et al. [5] and have been widely use for trigger point injection or needling. 

### 3.3. Other Types of Needling Therapies for Myofascial Pain

Fischer [22] suggested infiltrating with local anesthetic into the whole taut band including the myotendonal junction during MTrP injection, and performing “Preinjection blocks” to reduce the pain of needle penetration. Gunn used an AcP needle [23], and Chu [24] used an EMG needle, to perform dry needling to avoid the tissue damage by the sharp edge of the injecting needle. Furthermore, Chu et al. [25, 26] added electrical stimulation during treatment (“electrical twitch-obtaining intramuscular stimulation” or “ETOIMS”) in this technique, which is actually similar to electrical AcP. The technique of superficial dry needling (inserting the needle into the subcutaneous, but not the muscle tissues) has been demonstrated to be effective for treating myofascial pain [27–29]. Several authors have demonstrated the therapeutic effectiveness of pain control by MTrP injection with botulinum toxin A [30–32]. But the latest Cochrane review [33] suggested that there is inconclusive evidence to support the use of botulinum toxin in the treatment of myofascial pain syndrome.

### 3.4. Modified Acupuncture Therapy

Recently, Chou et al. [34] have developed a new technique of AcP therapy, which is similar to MTrP dry needling by insertion of the AcP needle into multiple sites of the MTrP region with a fast insertion speed (high pressure) to elicit LTRs. Simultaneous rotation of the needle (fast screwed-in and screwed-out technique) is also performed to facilitate the needle movement and to avoid bending of the small-sized AcP needle. In a related recent study on its therapeutic effectiveness, it was found that the irritability (measured as subjective pain intensity, pain threshold, and amplitude change of EPN) of the MTrP in the upper trapezius muscle could be suppressed after needling remote AcP points [35].

## 4. Probable Mechanism for Acupuncture in Pain Control

### 4.1. Chinese Traditional Acupuncture

Chinese traditional acupuncture is probably the first reported technique in treating patients with dry needling based on the Traditional Chinese Medicine (TCM) theory and has been commonly accepted for pain alleviation (analgesia) since 2500 years ago. TCM is a complex theory. In TCM concepts, the entire human body is composed of sophisticated interconnected inner systems, which there is an “energy (Qi)” that flows through “meridian (or channels)” in each organ [36, 37]. When the flow of Qi is blocked, pain and disease occur. By inserting and appropriate manipulating a needle into some points, the channel could be unblocked, thereby reestablishing the free and normal flow of Qi and relieving the pain. Most acupoints are located along one of these channels (some are exceptional). Diseases are caused by an imbalance or disturbance of Qi. Needling at the acupoints can harmonize Qi and treat diseases. These systems should be kept in balance to maintain a good health [36, 37]. These hypotheses, however, have not yet been validated by modern science and technology.

### 4.2. Acupuncture Analgesia

AcP has been widely used for treating patients with acute or chronic pain. Many comprehensive review articles on AcP analgesia have been published in recent years [38–41]. The mechanism of AcP analgesia has been widely explored since the 1970s, but still has not been adequately clarified. Many efforts have been made to clarify the mechanism of AcP analgesia, but no satisfactory consensus has been reached to date.

### 4.3. Endogenous Opiates (Morphine-Like Substrate) Theories

One Study for time-dependent analgesic effect in healthy humans was performed in the 1970s [42]. It showed that manual AcP at one hand acupoint induced a gradual increase in skin pain threshold and faded after removal of the needle. The effect was totally abolished after the infiltration of the local anesthetic procaine deep into the acupoint at the muscle and tendon layer, but not subcutaneously. The most well-known mechanism of AcP analgesia is the endogenous opiates (morphine-like substrate). Pomeranz and his colleagues [43, 44] have suggested that electroacupuncture (EAcP) induces endogenous opiates release from the pituitary gland into plasma and cause analgesia in the central nerve system. The followed researches disclosed that different kinds of endogenous opiates, such as *β*-endorphin [45, 46], enkephalin [46, 47], endomorphin [48], and dynorphin [49] also play very important roles for AcP analgesia. Cheng and Pomeranz [50] first described the possibility of different analgesic mechanism for different frequencies of EAcP. Later in Han JS's research group, they found that different types of opioid receptors mediated analgesia induced by EAcP of different frequencies [51]. More recently researchers claimed that the frequency is not the only determinate factor for analgesic effect. Lin et al. [52] found that intermittent-alternating mode of administering EAcP stimulation is a valid way to prevent tolerance. Lao and his colleague [53] systematically evaluated the antihyperalgesia of EAcP stimulation parameters (frequency, intensity, treatment duration, and pulse width) and suggested that the EAcP antihyperalgesia is parameter-dependent and point-specific. 

### 4.4. Serotoninergic Descending Pain Inhibitory Pathway Theories

In addition to the opioids, serotonin (5-HT, 5-hydroxytryptamine) had been speculated to be an analgesic transmitter in Cheng and Pomeranz's [50] early study and thought to play an important role in AcP analgesia. Tsai and his colleagues [54] conducted a rat study to find the possible mechanism of EAcP in reference to the effects of neuropeptides on serotonergic neurons and suggested that the influence of EAcP on 5-HT release may be due to activation of enkephalin-interneurons, which presynaptically inhibit the primary sensory neurons in the spinal cord. To elucidate which serotonin receptor subtypes involved in modulation of EAcP, Takagi and Yonehara [55] examined the inhibition effects of EAcP after different serotonin antagonists intravenous injected into rabbits and found that the 5-HT_2A_ receptor may be involved in modification of the transmission of the pain sensation through activation of excitatory pathways, and 5-HT_1A_ receptor activation may suppressively act on EAcP-induced analgesia. In a later study of Chang et al. [56], they found that the EAcP analgesia effect is blocked by 5-HT_1A_ and 5-HT_3_ antagonists at both low and high frequencies and enhanced by 5-HT_2_ antagonists at high frequency (100 Hz). Furthermore, serotoninergic descending pain inhibitory pathway theories have been also developed. There are many serotonin-releasing nuclei in the central nervous system. Raphe-spinal neurons were identified in the nucleus raphe magnus (NRM), which is one of serotoninergic neurons in the lower brainstem and the axons of them terminate at the level of spinal cord. Liu and his colleagues [57] found EAcP could activate these serotoninergic raphe-spinal neurons in the NRM, a supraspinal area mediating a negative feedback circuit of pain modulation, thus inducing analgesia via descending inhibition. It is also an excitatory receptor in the cortex and the hippocampus. Higher frequency EAcP might decrease the serotonin concentration within the cortex, acting as a sedative, then elicit analgesic effect via both the descending pain inhibitory pathway and the cortex [38].

### 4.5. Anti-Inflammatory Mechanism

In the 1990s, researchers conducted studies using inflammatory animal models [58, 59] and found AcP analgesia may be related to anti-inflammatory mechanism. More peptides (substance P, somatostatin and calcitonin gene-related peptide), which related to pain transmission, can be expressed after neurons have been injured [60]. With persistent inflammation, it can induce the hyperalgesia status (hyperexcitable to pain), then decrease pain threshold. If the peripheral tissues have been inflamed, some immune cells can excrete endogenous opiates that can bind with opioid receptors on peripheral afferent nerves [61], and then can inhibit the transmission of noxious signal from the peripheral nerve system to the central nerve system. In Sekido's study [58], they have suggested that peripheral opioid receptors are involved in EAcP analgesia during inflammatory conditions via local blockade instead of systemic blockade of opioid receptors. EAcP produces antinociception via release of endogenous opioid peptides, and then reducing the sign of hyperalgesia associated with inflammation [59]. 

### 4.6. Cholinergic Anti-Inflammatory Pathway

To date, there was debate about whether endogenous opiates act as neurotransmitters or hormones. Lin and Chen [38] have declaimed a “neuroimmune link”, which can answer for this argument well. Within central nerve system, endorphins are neurotransmitters, and within peripheral tissue, they are hormones. Inflammation is a local, protective response to microbial invasion or injury. The body's first defense against invading pathogens or tissue injury is the innate immune system. Tracey [62, 63] has reported that the cholinergic anti-inflammatory pathway is a neural mechanism that suppresses the innate inflammatory response. The nervous system reflexively regulates the inflammatory response and modulates immune responses in real time. This is the first time to postulate that the relationship between AcP and the anti-inflammatory reflex is mediated through the autonomic nerve system [62] and offer a unique opportunity to explore previously unrecognized techniques to treat disease and enable consideration of the neurological basis of complementary and alternative medical therapies, such as meditation and AcP [63]. 

### 4.7. Summary

According to these above researchers, Lin and Chen [38] have suggested that the analgesic effect of AcP is hypothesized to be related to immune, hormonal, and nervous systems. Compared to slow-acting hormonal system, nervous system acts in a faster manner. Given these complexities, AcP analgesia cannot be explained by only one single mechanism.

## 5. Needling (Including Acupuncture) for Pain from MTrPs—Clinical Trials and Basic Research 

### 5.1. Clinical Trials of Dry Needling for MTrPs

Although the analgesic mechanism of dry needling (including AcP) effect is still not well known, the practice of inserting needles into soft tissue tender points to alleviate pain is well accepted. The clinicians commonly adopting the orthodox of injection, dry needling, or the traditional AcP. In one of the early studies investigating dry needling for trigger point, Lewit [64] conducted a study investigating short- and long-term effects of dry needling in the treatment of chronic myofascial pain. Effectiveness for alleviating chronic myofascial pain was noted, and the immediate analgesia produced by needling the pain spot has been called the “needle effect”. It is still unclear if the MTrP injection can also provide significant needle effect. Many authorities strongly believe this possibility [7, 65, 66]. 

Cummings and White [67] conducted a systematic review of needling therapies in the management of MTrP pain. Marked improvements occurred in all groups in which trigger points were directly needled, but the authors concluded that the hypothesis that needling therapies have efficacy beyond placebo is neither supported nor refuted by the evidence from clinical trials. A recent systematic review [68] identified 26-randomized control trials of AcP and dry needling in the management of MTrP pain after searching electronic database. Only seven studies were included, but needling was not found to be significantly superior to placebo after a meta-analysis. They have concluded that there is limited evidence deriving from one study that deep needling directly into MTrPs has an overall treatment effect when compared with standardized care. They have also suggested that the limited sample size and poor quality of these studies highlight and support the need for large scale, good quality placebo controlled trials in this area.

Objective evaluation of the effectiveness of trigger point acupuncture on pain and quality of life in chronic neck pain patients compared to three other AcP treatments (acupoints, nontrigger point and sham treatment) was conducted by Itoh and his colleagues [69]. They found that the trigger point AcP group reported less pain intensity and improved quality of life compared to the sham or nontrigger point groups after treatment, and suggested that trigger point AcP therapy might be more effective on chronic neck pain in aged patients than the standard AcP therapy. In a more recent study, Sun et al. [70] investigated the effects of AcP on patients with chronic neck myofascial pain syndrome by a single-blind randomized controlled trial and found that AcP group had greater improvement in physical functioning and role emotional of Short Form-36 quality of life than the control group at 12 weeks followup. 

### 5.2. Basic Researches of Dry Needling for MTrPs

It has been suggested that LTR should be elicited during dry needling in treating MTrPs [65, 71]. An animal model for myofascial pain study was initially reported in 1994 [71]. A sensitive spot could be found in the biceps femoris muscle of a rabbit, which defined as a “myofascial trigger spot (MTrS)”. When this sensitive spot was compressed before anesthesia, the rabbit screamed and kicked with an expression as if suffering very painful stimuli. This spot was marked and the animal was anesthetized for further study. This rabbit MTrS model provided at least three important characteristics similar to human MTrP: pain, LTR, and spontaneous electrical activity (SEA) and can be reasonably accepted for scientific research [71]. Local twitch response, a sudden brisk powerful contraction of a group of muscle fibers, could be elicited by needle stimuli in the MTrS [71]. Both LTR and SEA were suppressed after repeated needling on the same locus in the rabbit MTrS region [72]. It appears that, after a sensitive locus is encountered, and an LTR is elicited, the irritability of this sensitive locus (nociceptors) can be suppressed. 

It has been suggested that LTR is an involuntary spinal reflex contraction of muscle fibers within a taut band, and occurs during needling of a taut band associated with pain relief and reduction of stiffness [73]. Shah and his colleagues [74, 75] developed a microanalytical system, including a small size and shape needle (as an AcP needle) to simultaneous sampling of skeletal muscle tissue to analysis the biochemical milieu of substances related to pain and inflammation. They found the concentrations of substance P (SP), calcitonin gene-related peptide (CGRP), bradykinin (BK), 5-hydroxytryptamin/serotonin (5-HT), norepinephrine (NE), tumor necrosis factor alpha (TNF-*α*), and Interleukin 1-beta (IL 1-*β*) were higher in the active MTrP group than in the latent MTrP and no-MTrP groups before elicited LTR. In the active MTrP group, the SP and CGRP concentrations post-LTR, corresponding to clinically observed decrease in pain and tenderness after MTrP release by dry needling were significantly lower than the pre-LTR values with compared in the latent MTrP and no-MTrP groups. 

### 5.3. Probable Mechanism of Dry Needling for MTrPs

The most likely mechanism of pain relief by needle stimulation is hyperstimulation analgesia [76] via the descending pain inhibitory system. Melzack's gate control theory of pain describes the modulation of sensory nerve impulses by inhibitory mechanisms in the central nervous system [76]. The strong pressure stimulation to the MTrP loci can provide very strong neural impulses to the dorsal horn cells in the spinal cord, which may then break the vicious cycle of the MTrP circuit [2, 8]. The fast needle movement technique developed by Hong [21] can provide high-pressure stimulation, then inactive the MTrPs [5, 77]. Effective inactivation of the MTrP can relieve the pain and uncomfortable symptom [2, 10, 22, 65, 66]. The mechanism of local effects on the site (MTrP) of needling for the immediate relief of pain after AcP or dry needling has been consider to be mediated via the neural pathway [27, 77].

Gerwin et al. [78] claimed that the muscle soreness and pain associated with MPS is related to the chemical activation of nociceptors by substances released from surrounding injured tissue, then stimulate a unique cascade of cytokines that are integral to the inflammatory response. There is a biochemical basis to the development of peripheral and central sensitization in muscle pain while AcP (or dry needling) to the MTrP. In recent Shah's researches [74, 75], they confirmed that biochemicals associated with pain, inflammation, and intercellular signaling (e.g., inflammatory mediators, neuropeptides, catecholamines, and cytokines) were elevated near the active MTrPs differentiated from latent MTrPs and muscle without MTrPs. Assaying the peripheral milieu of a MTrP before, during, and after an LTR could disclose changes in bioactive substances that may contribute to myofascial pain. These initial peripheral conditions within muscle seem to be the source of feed-forward mechanisms that transform and intensify central processing of muscle pain.

## 6. Clinical Studies of Remote Needling (Including Acupuncture) for MTrP 

### 6.1. Selection of Acupoints Based on TCM Principles

AcP on the tender points has been commonly used as a treatment for chronic musculoskeletal pain and appears to alleviate pain and stiffness in clinical practice. These tender points are known to be located at traditional acupoints, “*Ah-Shi*” points. They are supposed to be the sites where nociceptors and polymodal receptors, could be sensitized by various factors. Thus, AcP on the tender points may activate sensitized polymodal receptors and result in stronger effects on pain relief [79]. But, AcP is not only applied to the “*Ah-Shi* ” point but also to the distal acupoints for the treatment of chronic pain. 

In the view of TCM, the Huang di nei jing (Yellow Emperor's Inner Canon) [36, 37] states that, “if someone has disease related with the left side, the treatment point is the right side, and vice versa,”emphasizing the importance of treatment side. In Han's review article, he suggested that there seem to be at least three principles for selection of acupoints based on the TCM principles. (a) “It is more important to identify the right meridian rather than the right point within the meridian”. (b) “To use the meridian with its pathway reaching the vicinity of the diseased organ” (c) “Where there is pain, there is a transport point” (“*Ah-Shi*” point) [41]. 

Cheng [80] reviewed the AcP treatment formulae for some common conditions and found that the acupoints traditionally used for the treatment have a neuroanatomical significance from the viewpoint of Western medicine in many cases. These mechanisms of action include intramuscular stimulation for treating muscular pain and nerve stimulation for treating neuropathies. In Cheng's later study [81], he examined the relationship between the anatomical location of traditional acupoints and their clinical indications as stated in two textbooks of TCM and concluded that (1) the acupoints in the trunk and their stated effects on the internal organs in the trunk have a segmental relationship, (2) the acupoints in the trunk and extremities have a musculoskeletal effect that is local or regional, but not distal, and (3) the acupoints on the head and neck preferentially affect the nearest organ. 

### 6.2. Effectiveness of Needling at Distant Points for MTrP

Some clinical trial studies showing that AcP's analgesic effect can reach distant sites in the recent research. Irnich and his colleagues evaluated immediate effects of needle AcP at distant points and dry needling of local MTrPs on motion-related pain and cervical spine mobility in chronic neck pain patients compared to a sham laser AcP. Their results indicated that AcP has specific effects on motion-related pain and range of motion in patients with chronic neck pain. Needling at distant points improving range of motion more than at local points, and local points seeming ineffective to obtain immediate relief of motion-related pain [82]. Xue et al. conducted a randomized, single-blinded, sham-controlled, crossover clinical trial to investigate the efficacy of EAcP, applied to distal acupoints only, for tension-type headache and found it is effective for short-term symptomatic relief of tension-type headache [83]. 

Hsieh and her colleagues [73] conducted a clinical study and provided evidence that dry needling-evoked inactivation of a primary (key) MTrP (in the infraspinatus muscle) inhibits the activity in satellite MTrPs (in the ipsilateral anterior deltoid and extensor carpi radialis longus muscles) situated in its referral pain zone. In the recent study of Tsai et al. [84], they found dry needling of a distal MTrP (in the extensor carpi radialis longus muscle) could provide a remote effect to reduce the irritability of a proximal MTrP (in the upper trapezius muscle). Matsubara and her colleagues [85] had investigated the effect of acupressure at local and distal acupoints in females with chronic neck pain, and found pain-related condition (like verbal rating scale, neck disability index, State-Trait anxiety inventory, and muscle hardness) improved not only the local points but also the distal acupoints with compared with control group.

The clinical implications of these above studies may involve the selection of remote acupoints for treating MPS patients, which is thought to be one of the key issues in therapeutic effectiveness of AcP. But the selective outcome assessments in these researches are all subjective, like verbal rating scale and questionnaire, and some semisubjective, like pain pressure threshold obtained via algometer. Some objective assessment is needed.

### 6.3. Correlation between Acupoints and MTrPs

Important characteristics of an MTrP include local pain or tenderness, referred pain or referred tenderness, and local twitch response. Melzack et al. [86] published the first review article in discussion with correlations and implication between trigger point and acupoints for pain. He found the trigger points are firmly anchored in the anatomy of the neural and muscular systems, while acupoints are associated with an ancient conceptual but anatomically nonexistent system of meridians. He suggested a hypothesis that trigger points and acupoints for pain, though discovered independently and labeled differently, represent the same phenomenon and concluded a remarkably high degree (71%) of correspondence with MTrPs and acupoints [86]. Hong had concluded that there are many similarities between acupoints and MTrPs including their location and distribution, pain and referred pain patterns, local twitch responses (*De-Qi*), and so forth [77]. 

Travell and Simons [1] said: “acupuncture points and trigger points are derived from vastly different concepts. The fact that a number of pain points overlap does not change that basic difference. The two terms should not be used interchangeably”. Birch [87] revisited trigger point-AcP point correlations through a more extensive examination of the AcP literature. He claimed that the findings of Melzack's 1977 study appear to be incorrect, 71% correspondence of trigger points to acupoints is conceptually not possible. He suggested that some overlap in the location of acupoints and trigger points is possible, but unlikely to be more than chance (not more than 40%, probably closer to 18%), and such similarity of location does not imply a correlation [87]. Beside, Birch found trigger points are a much better match to the class of acupoints called “*Ah-shi*” points than to the “channel” and “extra” acupoints in the treatment of pain and approximately 35% of recommended acupoints in the treatment of pain are distant from the site of the pain [87]. 

Dorsher [88] comprehensively examined the anatomic and clinical relationships between each trigger point described by Travell and Simons [1, 89] and the classical, “miscellaneous,” and “new” acupoints described by the Shanghai College of Traditional Medicine [90]. A total of 255 trigger points were compared with 747 acupoints. He found strong anatomic (92%), clinical (79.5%), and meridian-referred pain (76%) correspondences of trigger and acupoints in this study and suggested AcP meridians were shown not only to exist conceptually but also, to be physiologic (and possibly anatomic) entities. In the later study, Dorsher [91] reviewed AcP references and literature to re-examine the validity of the findings by Birch in 2003 [87]. He suggested the conceptual comparison of trigger points to classical acupoints in pain disorders treatment, and their clinical correspondence in this regard is likely 95% or higher. The AcP and myofascial pain traditions have fundamental clinical similarities in the treatment of pain disorders and myofascial pain data and research may help elucidate the mechanisms of AcP's effects [91]. 

### 6.4. Objective Assessment for the Remote Effect of Dry Needling (Acupuncture) for MTrP

SEA, include EPN and endplate spike are usually observed in an MTrP region in human. The prevalence of EPN [92] or the amplitude of EPN [35] is proportionate to the irritability of a MTrP. Current evidence shows that SEA at MTrP originates from the extrafusal motor endplate [93], and Ge et al. [94] concluded that SEA at MTPs might play a significant role in the induction of pain.

In the recent studies of Chou et al. [95], they chose the changes of mean EPN amplitude for the objective outcome assessment and found the MTrP irritability in the upper trapezius muscle could be suppressed after a remote AcP treatment on both *Wai-guan* and *Qu-chi* acupoints. Beside, needling to the remote acupoints with multiple needle insertions of modified AcP technique seems better than simple needling insertion and sham AcP.

## 7. Animal Studies of Remote Needling (Including Acupuncture) for MTrP 

Referred tenderness is a phenomenon of remote referral of pain in response to compression of an MTrP, while referred pain is spontaneous pain referred to the remote sites from an MTrP [5]. The occurrence of referred tenderness and/or pain depends on the irritability of the MTrP and the pressure of compression [3, 96, 97]. For the MTrP in a certain muscle, the referral pain area locates within the same territory consistently if it is elicited at different time on the same person or at any time in different persons [1, 89]. 

In those previous researches, Mense et al. have demonstrated the referred pain from a muscle to another distant muscle in animal studies [98–100]. Pain signals from peripheral stimuli could be electrophysiologically recorded from sensory neurons in the dorsal horn of the spinal cord in a rat. For a dorsal horn neuron, mapping various sites receiving stimuli to elicit responses of this dorsal horn neuron can identify its receptive field. Later, Mense and Simons [101] conducted an animal study on the referred pain from a muscle to other distant ones. They suggested that strong noxious stimulus could send the impulse to and induce the corresponding dorsal horn neuron release substance P and calcitonin gene-related peptide (CGRP). Then increase the efficacy of silent synaptic connections as a consequence of central sensitization in the spinal cord level.

Sluka and colleagues [102] developed a model of musculosckeletal pain. Repeated injection of low pH saline into the gastronemius muscle and a long lasting, widespread mechanical hyperalgesia without motor deficits was produced. Contralateral decrease in mechanical threshold was noted and suggested centrally mediated hyperalgesia. In Sato's recent studies [103] of the neural mechanisms of the reflex effects on visceral functions of AcP-like stimulation applied to the skin and underlying muscle by twisting a needle in anesthetized rats, they have identified a wide variety of reflex responses in remote modification of various organ functions. 

Hsieh's more recent study [104] is the first animal study to investigate the nueral mechanism of the remote effects of dry needling. As demonstrated in their study, the irritability of MTrSs at biceps femoris muscle (proximal MTrS) could be modulated by the remote effect of dry needling either ipsilaterally or contralaterally at MTrS of gastrocnemius muscle (distant MTrSs). After the transection at either ipsilateral tibial nerve or L5-L6 segments, loss of this remote effect on the change of EPN amplitude at MTrS of biceps femoris muscle was note, but not at T1-T2 segments. They have found that the neural pathway of the remote needling effect is mediated via an intact afferent connection from the site of stimulation to the spinal cord and a normal spinal cord function at the level corresponding to the innervations of the responded remote muscle. They further suggested that the influences from higher supraspinal level such brainstem, midbrain structures involved in the descending pain inhibitory system needs further investigation. 

## 8. Possible Mechanism for Remote Needling (Including Acupuncture) of MTrP 

The descending pain inhibitory system [38, 105–107] is probably involved in the mechanism of immediate pain relief due to needling (including acupuncture). Either hyperstimulation analgesia for general pain control [76] or disruption of “MTrP” circuit for myofascial pain control [2, 8, 104] as a proposed mechanism of needling effect for pain relief is actually via the descending inhibitory system. 

A low-pressure stimulation to a sensitive locus may elicit a local pain only. When the stimulation force is increased progressively, the subject who has been needled may feel increased of pain intensity, and then may feel referred pain, and finally LTR can be elicited. Immediate relief of MTrP pain can be expected if LTRs are elicited during needling of the MTrP [21, 65, 108, 109], and the irritability of this sensitive locus can be suppressed [35, 92]. When a LTR is elicited, the patient usually feels a sharp pain with referred pain and muscle twitching. Such feeling is similar to that when a “*De-Qi*” effect is obtained during AcP therapy. 

Regarding the mechanism of remote needling (AcP) effects to inactivate an MTrP, it is probably related to a spinal cord mechanism similar to the MTrP mechanism [3, 77]. It is possible that activation of nociceptors in the skin or muscles by needle stimulation (high pressure) can send strong sensory impulses to the spinal cord or higher center to interfere with all the related “neural circuits” of pain modulation (similar to “MTrP circuits” described by Hong) [2, 8]. Furthermore, AcP (needling) to a key MTrP can also inhibit the irritability of satellite MTrPs [73] due to central desensitization phenomenon.

In Cheng's study [81], he concluded acupoints within certain spinal segments in the trunk affect the functioning of the organs that receive autonomic innervations from the same spinal segments. This is consistent with the concept of segmental AcP and the idea that AcP may act via the somatic sympathetic reflex with a spinal pathway to affect the trunk organs. Hsieh et al. [104] suggested that an intact afferent nerve from the remote stimulation site and normal spinal cord segments corresponding to the innervation of the affected proximal muscle are essential for the remote effect from either ipsilateral or contralateral needling stimulation. This neural pathway of remote effectiveness is probably mediated via a spinal reflex similar to that mediated the LTR [71, 110] and probably also similar to that for the referred pain. The physiological basis for the remote effects of AcP therapy may relate to a consequence of diffuse noxious inhibitory control that induced by noxious stimulation applied at the painful region (such as trigger point needling) or at a remote site (such as in remote AcP or dry needling) [111, 112].

## 9. Conclusion

Myofascial pain is one of the most common problems of musculoskeletal pain and is treated with a variety of conservative and invasive therapies. Previous clinical and animal investigations on the MTrP treatments focused on the existence of taut band, LTR, and referral pain. Tender points or MTrPs as “*Ah-Shi*” points are frequently used in AcP for MPS pain control. Recently, remote effectiveness of AcP (or dry needling) has been emphasized. 

Although the specific pathophysiological basis of MTrP development is still not completely clear, several promising scientific studies (i.e., electrophysiological, neurophysiological, and biochemical) have revealed objective abnormalities. These findings suggest that myofascial pain is a complex form of neuromuscular dysfunction consisting of motor and sensory abnormalities involving both the peripheral and central nervous systems, and needling therapy can provide significant pain relief via neural mechanism.
